# Supplementary material for: Single-cell lineage tracing in the mammary gland reveals stochastic clonal dispersion of stem/progenitor cell progeny
Source: Nat Commun. 2016 Oct 25;7:13053. doi: 10.1038/ncomms13053 (PMC5093309; doi:10.1038/ncomms13053)
Supplement: Supplementary Information — Supplementary Figures 1-14 [file ncomms13053-s1.pdf]

a

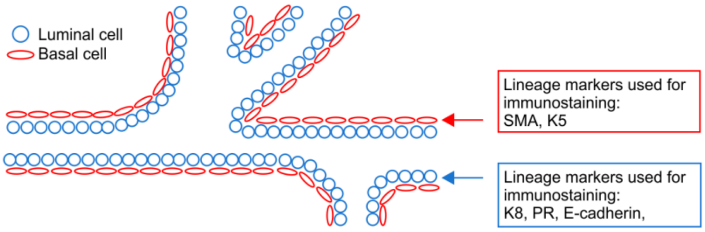

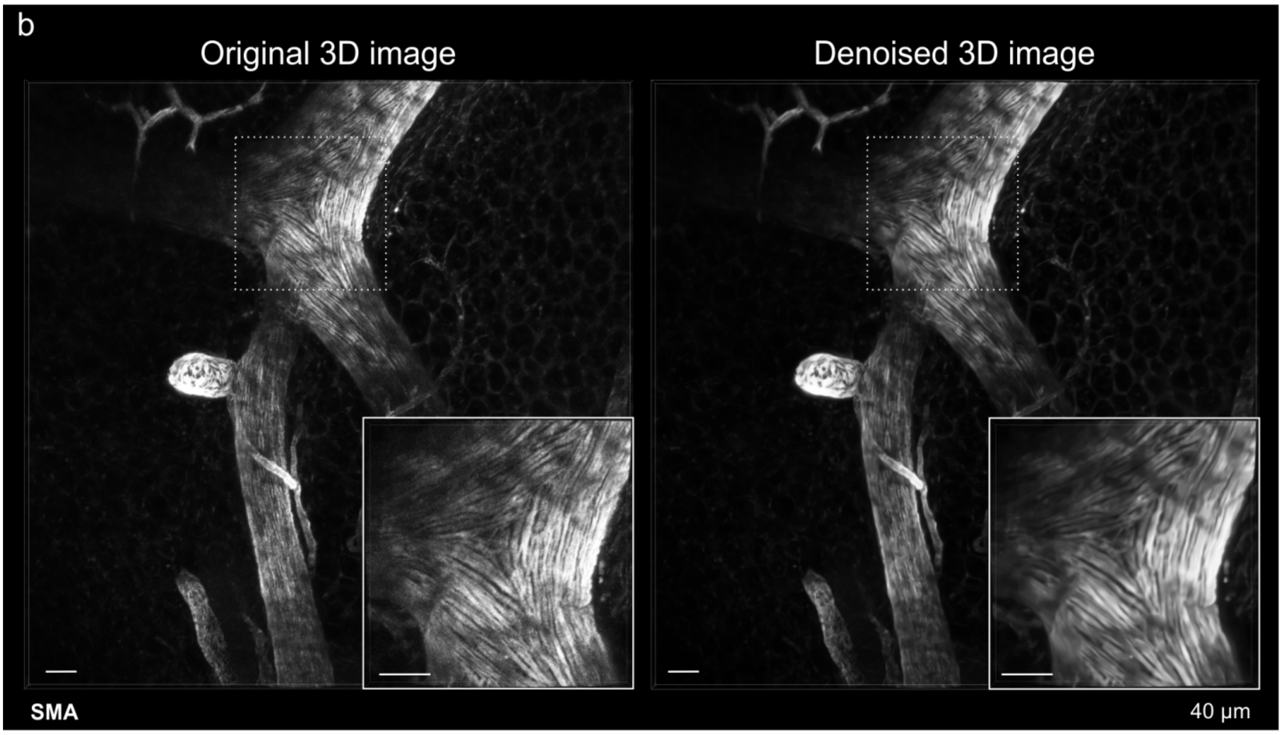

Supplementary Figure 1: 3D imaging and post-processing. a, A key to the lineage markers used in this study. **b**, Example of SMA immunostaining in virgin mammary tissue before and after a denoising algorithm was applied to minimise Poisson-Gaussian noise in 3D image stacks.

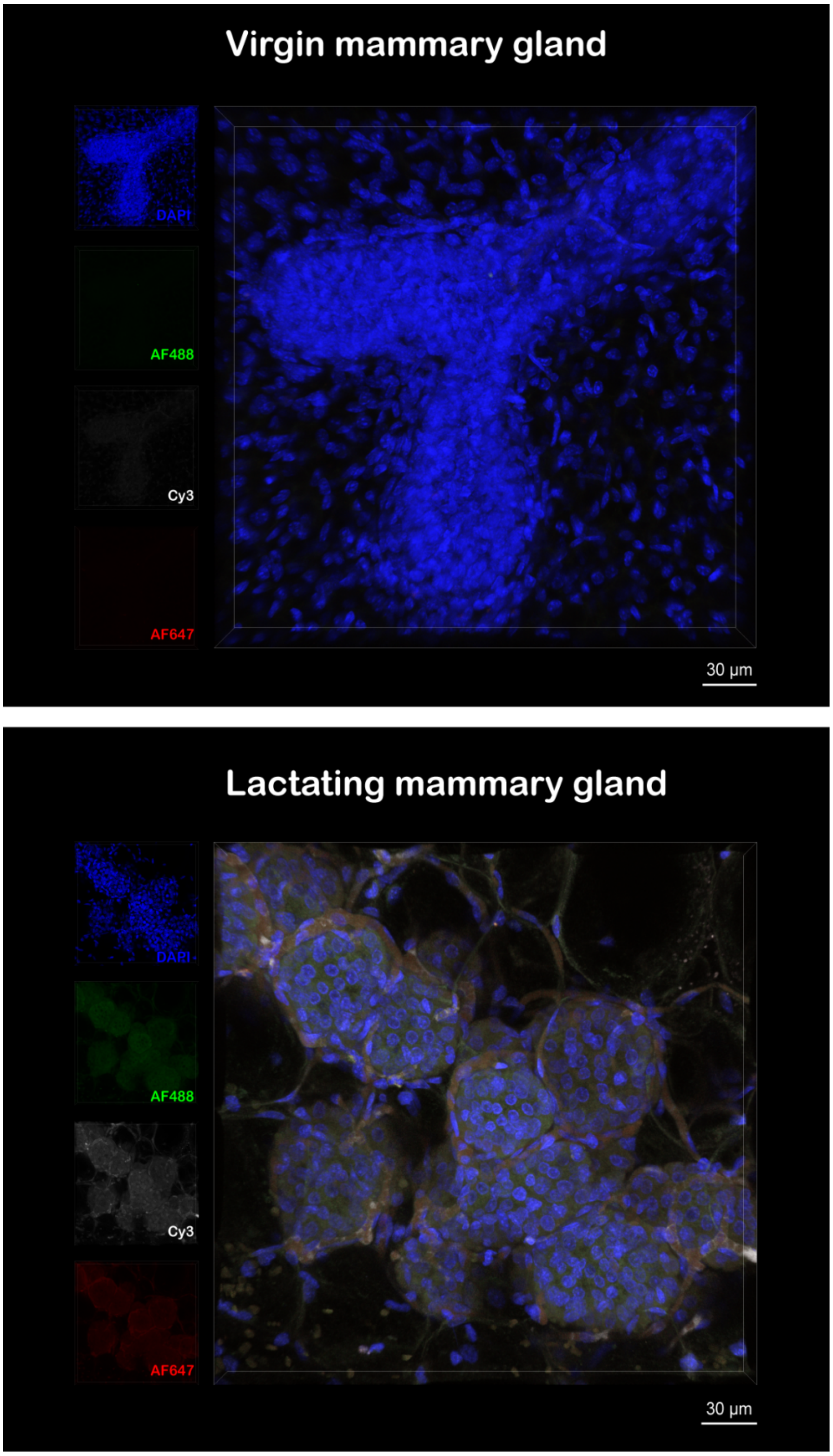

Supplementary Figure 2: Control image sequences of tissue from virgin and lactating mice. No primary antibody controls.

a

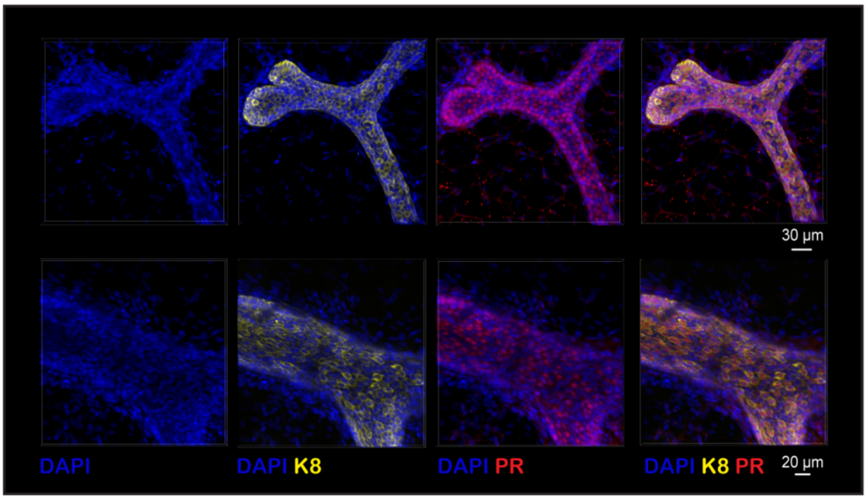

b

	Average ± S.D.
PR^{+} cells that are $K8^{hi}$	79.95 ± 8.10%

c

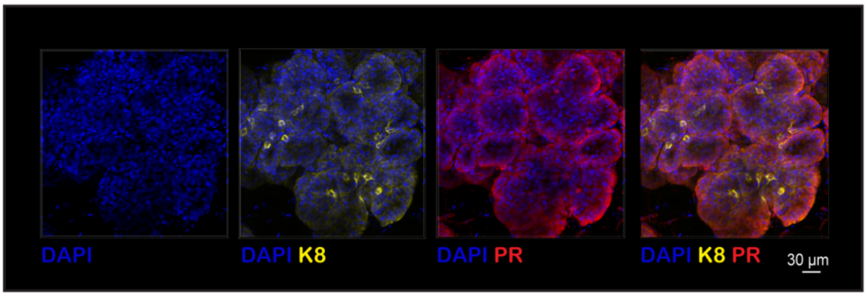

Supplementary Figure 3: $K8^{hi}$ cells are associated with the hormone receptor positive luminal lineage. **a**, Representative 3D confocal images showing non-uniform expression of K8 in luminal cells, with $K8^{hi}$ cells co-staining with nuclear progesterone receptor (PR). **b**, Table showing the proportion of PR positive cells scored as $K8^{hi}$ in virgin pubertal mammary glands. Data represent the quantification of 6 images from 3 mice (1831 cells scored in total). **c**, Rare $K8^{hi}$ /PR^{+} luminal alveolar cells are observed in lactating mammary tissue.

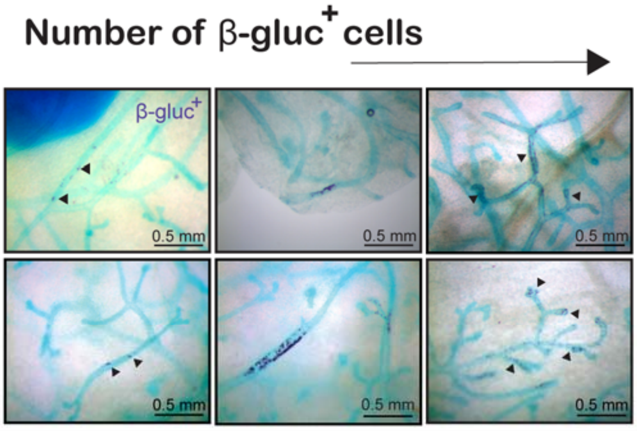

Clone size	Small	Moderate	Large
Average no. per gland	0.57 ± 0.69	0.82 ± 0.27	0.10 ± 0.08
Total		1.49 ± 0.92	

Supplementary Figure 4: Rate of strand slippage in the mammary gland of $R26^{[CA]30SYNbglA}$ pubertal mice. The event rate in the mammary gland was determined using $R26^{[CA]30SYNbglA}$ mice, as this model is conducive to macroscopic analysis of all labelling events. Examples of small (approx. 2-5 cells), moderate (approx. 5-50 cells) and large (> 50 cells) β-glucosidase$^+$ regions are also shown. This last category includes those regions thought to have arisen from a MaSC based on size and number of branches, which occurred at a rate of approximately 0.03 ± 0.06 per gland. Labelling was stochastic and thus associated with a large error. Values show average ± S.D. quantified from 9 pubertal hemizygous mice.

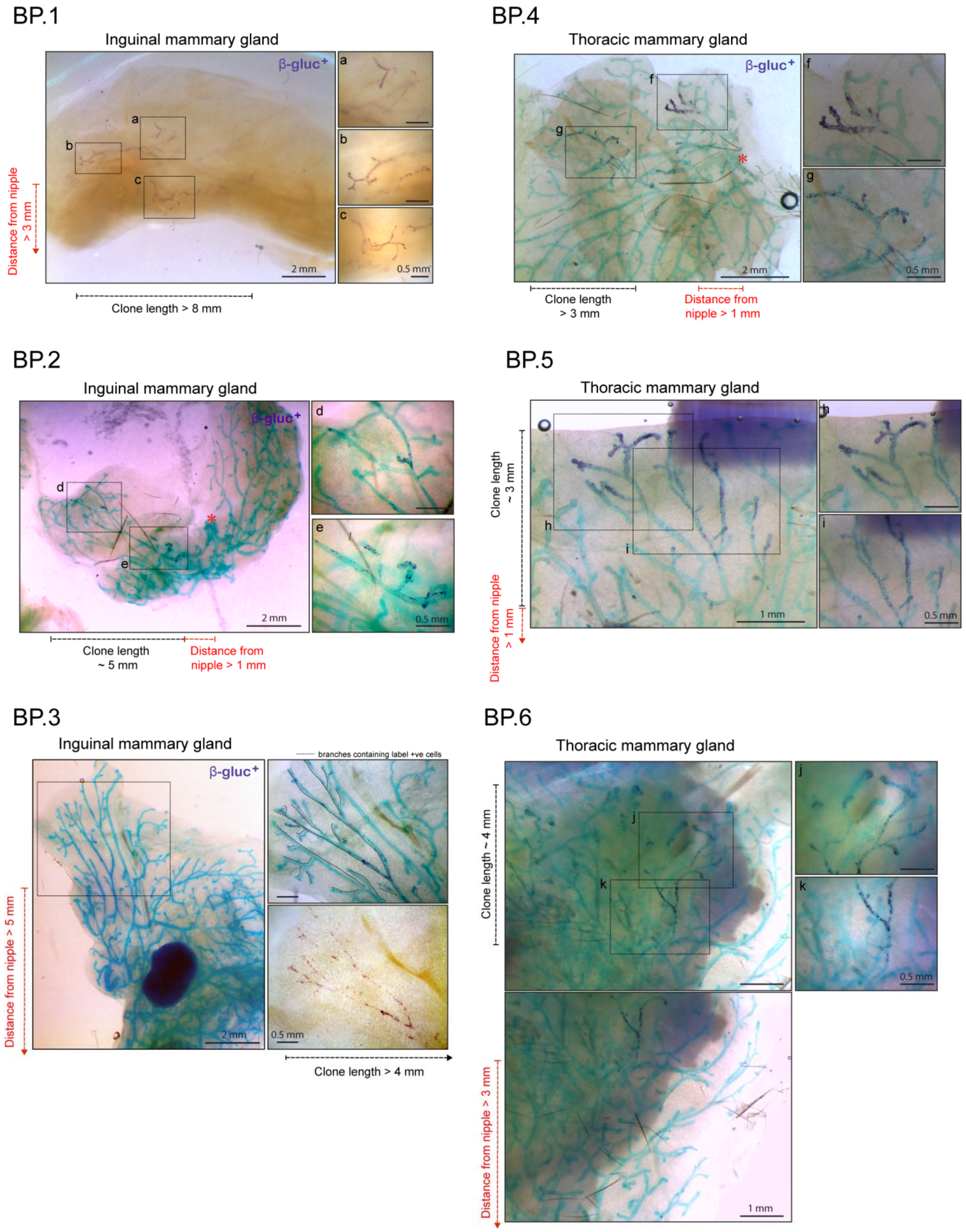

Supplementary Figure 5: Clone size and labelling patterns arising from β-glucosidase^{+} MaSC/progenitor clones related to Figure 2d. Wholemount transmission images showing the clonal labelling patterns (β-glucosidase^{+} cells interspersed with unlabelled cells), clone size and approximate distance from nipple (asterisk) of clones BP.1 to BP.6.

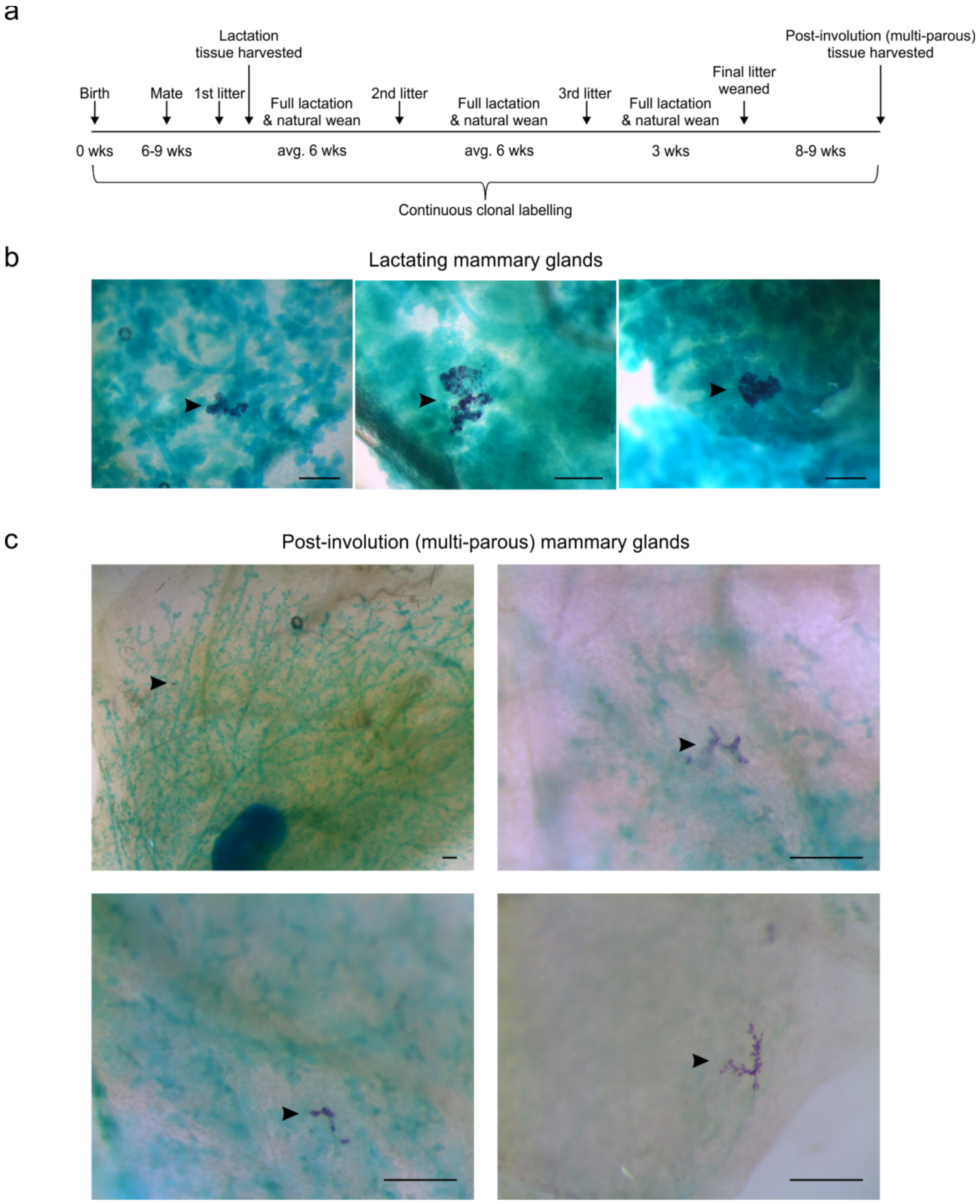

Supplementary Figure 6: β-glucosidase⁺ cells can be observed in *R26*$^{[CA]30SYNbglA}$ mammary glands after multiple pregnancy/involution cycles. a, The experimental protocol for obtaining lactating (**b**) and multiparous (**c**) mammary tissue. Mice of reproductive age were mated and lactating tissue was harvested from some mice on lactation day 4. A cohort of mice were taken through three pregnancies each followed by a natural (approx. 20 day) wean. Mammary tissue was harvested 8-9 weeks after the final wean. While these β-glucosidase^{+} cells (purple, arrowhead) may represent the progeny of a long-lived MaSC, since this is a continuous clonal labelling model, the originating cell may have been labelled at any stage of the study (approx. duration 8 months). Scale bar shows 0.5 mm. n = 3 mice for each time-point.

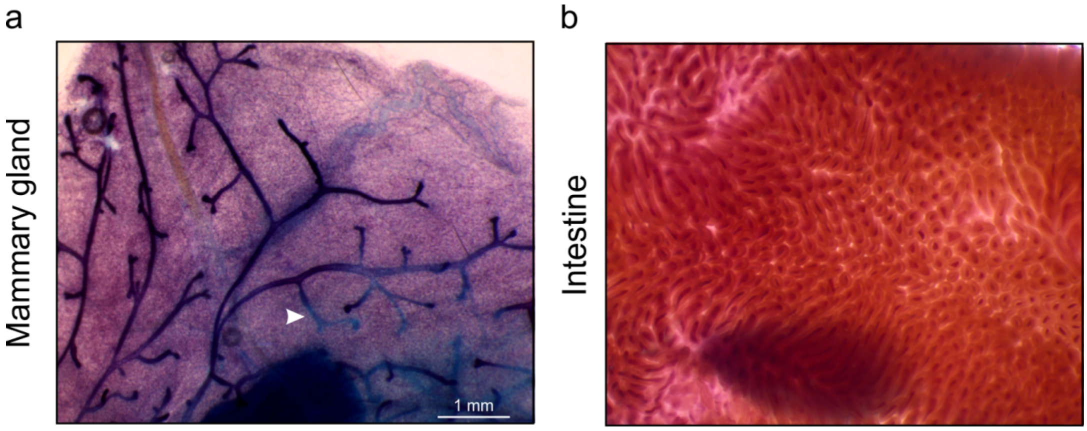

Supplementary Figure 7: Mammary and intestinal tissue from a mouse with ubiquitous β-glucosidase reporter expression due to a germ line $[CA]_{31}$ mutation. a, β-glucosidase reporter expression (purple) in mammary tissue from this germ line mouse confirms complete and uniform reporter expression in luminal and basal mammary epithelial cells, as well as stromal cells. Mammary gland wholemounts were counterstained with methyl green. Arrowhead shows region that did not stain purple due to exhaustion of the colorimetric substrate for β-glucosidase. **b**, Uniform β-glucosidase expression in intestinal tissue confirms the germ line $[CA]_{31}$ mutation.

Clone YP.2

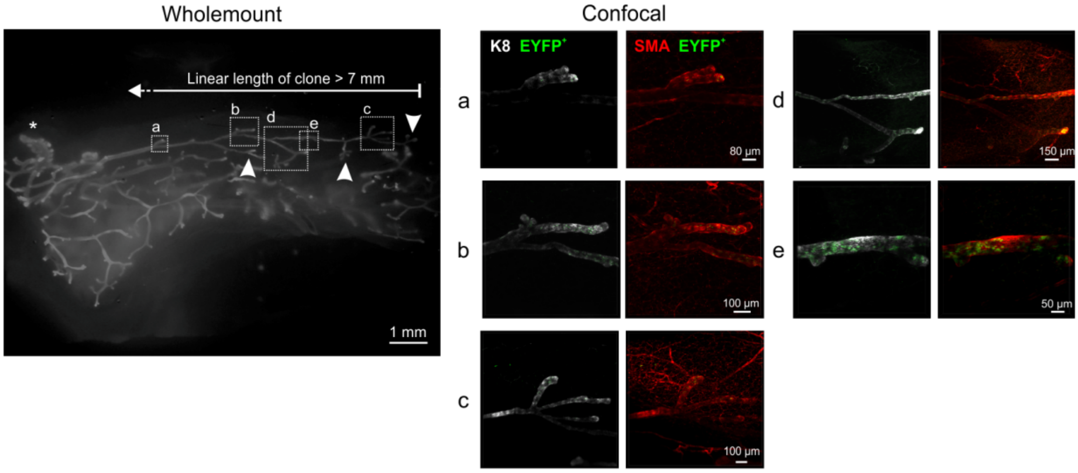

Additional distinct EYFP+ region observed in the same mammary gland as clone YP.3

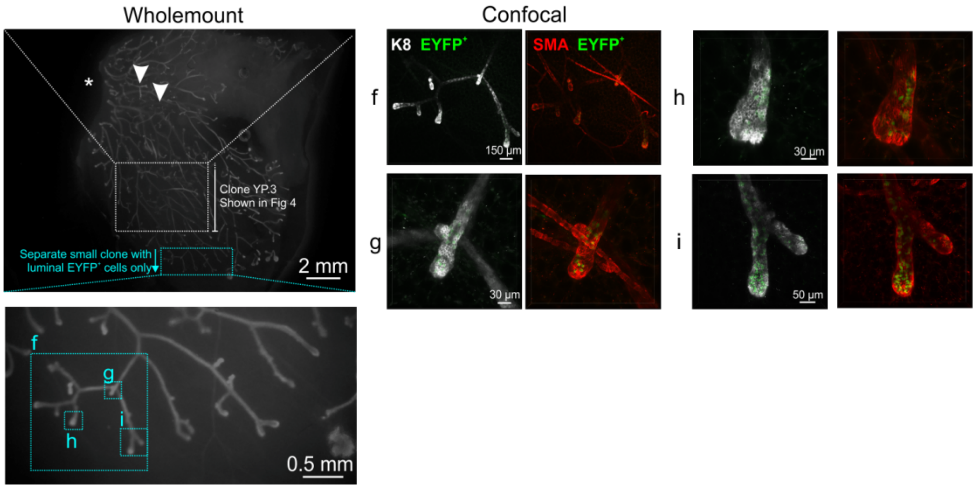

Supplementary Figure 8: Clone size and labelling patterns arising from EYFP$^+$ presumptive MaSC clone YP.2 and an additional region observed adjacent to clone YP.3. Wholemount fluorescence image (K8 immunofluorescence) of the mammary ductal network, mapping regions (**a-e**), spanning over 7 mm in linear length, that were imaged using a confocal microscope. All EYFP$^+$ cells in clone YP.2 were luminal. Wholemount fluorescence images (K8 immunofluorescence) of the mammary ductal network, showing region (white box) containing EYFP$^+$ basal cells (mapped in Fig. 4, clone YP.3). A separate region was identified in this tissue piece from this continuous clonal labelling model that contained only luminal cells (blue box, regions (**f-i**)). Luminal and basal EYFP$^+$ cells were not observed in the same branches and were never intermixed. Asterisks show the nipple and origin of the ductal network. Arrows show regions identified on the fluorescence stereoscope that were thought to contain a few EYFP$^+$ cells that were unable to be imaged on the confocal microscope due to their depth.

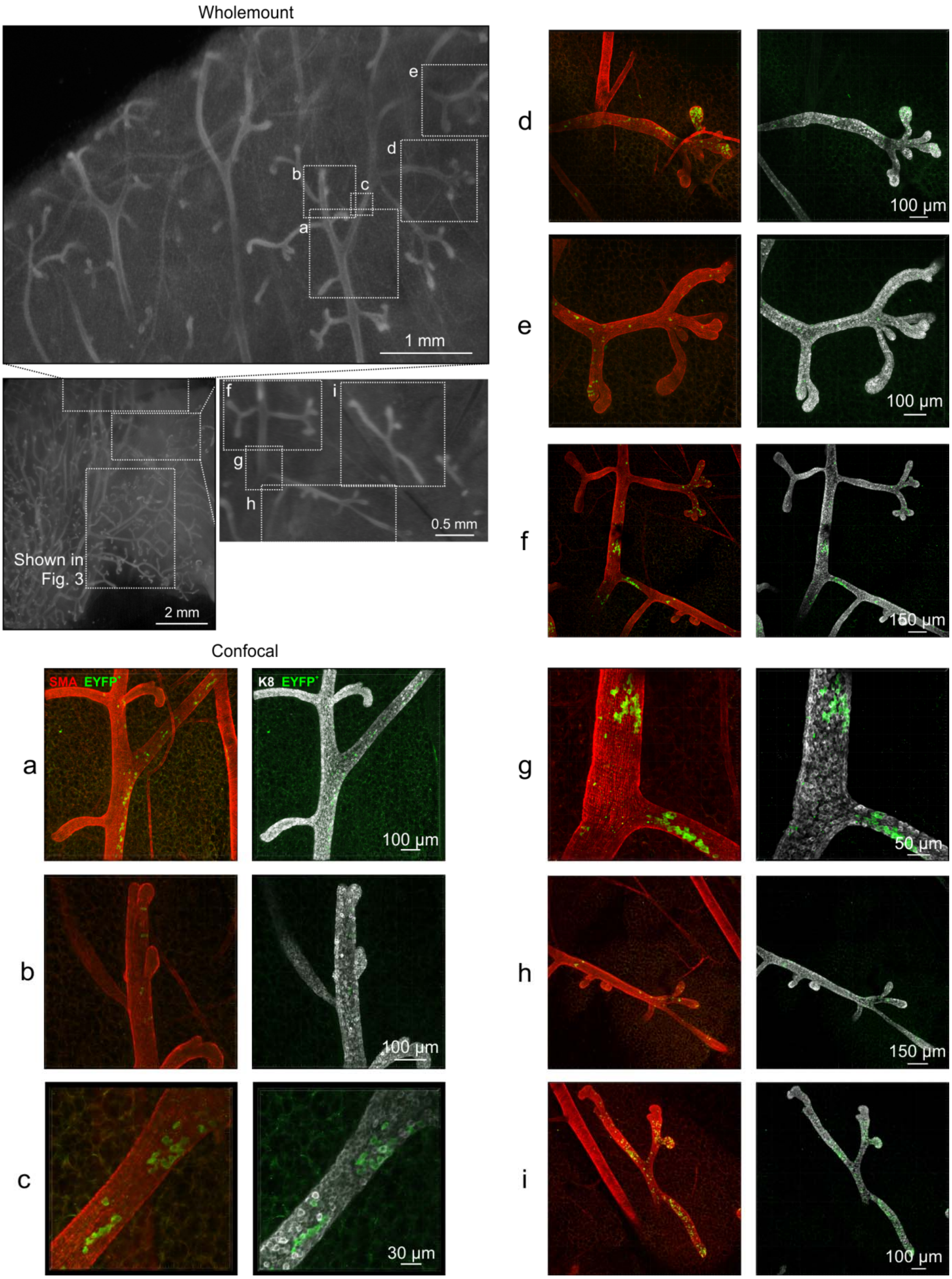

Supplementary Figure 9: Clone size and labelling patterns arising from a single luminal EYFP$^+$ presumptive MaSC related to Figure 3. Wholemount fluorescence images (K8 immunofluorescence and DAPI) of the mammary ductal network, mapping regions (**a-i**) that were imaged at high spatial resolution using a confocal microscope. All EYFP$^+$ cells in this clone (YP.1) had a luminal morphology, expressed K8 and were negative for SMA. Additional regions from this clone, which spanned > 8 mm in length, are shown in Fig. 3.

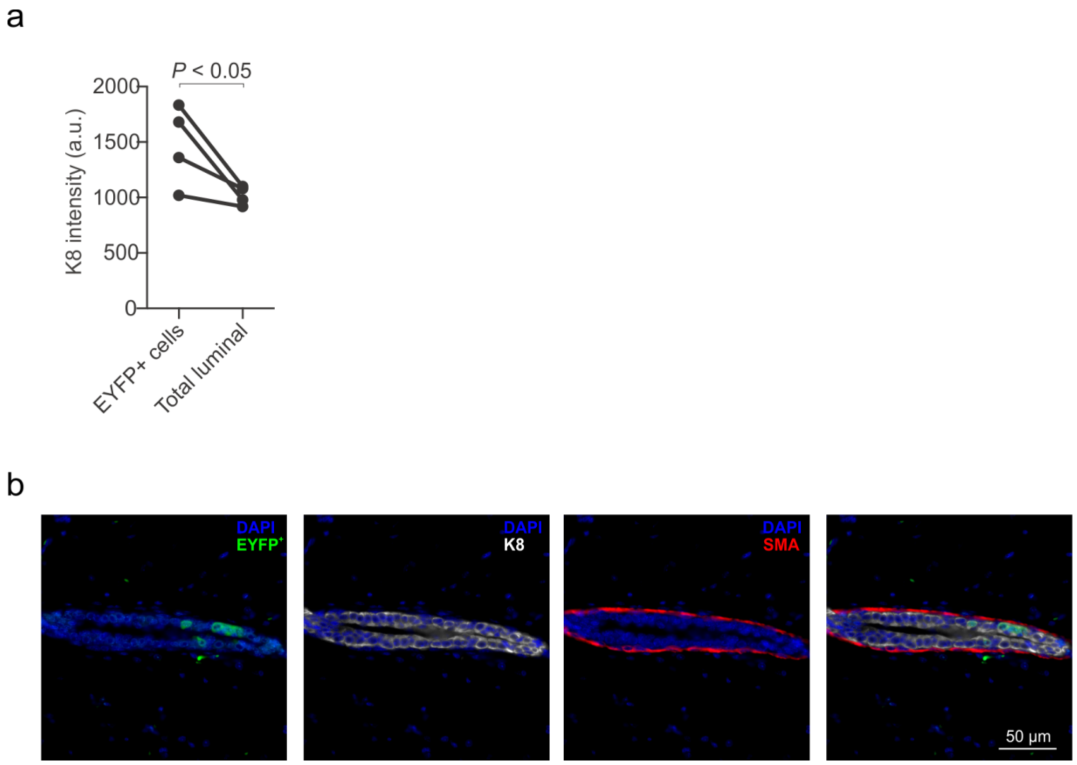

Supplementary Figure 10: 2D and 3D analyses of a clone arising from a single EYFP$^+$ cell related to Figure 3. a, To determine whether a single marked luminal MaSC/progenitor contributed equally to both K8^{hi}/PR$^+$ and K8^{lo}/PR$^-$ populations we analysed a clonal region (YP.1) using custom written 3D imaging algorithms (see Methods). A *Kolmogorov-Smirnov* test was performed to determine if the significant voxels of the segmented total ductal luminal cells were differently distributed to the significant voxels inside segmented EYFP$^+$ cells. In each of the four cases analysed within a single clonal region the null hypothesis of equality of the two distributions was rejected (at P = 0.05), revealing that the K8 signal inside the EYFP$^+$ cell was significantly higher than the total luminal population. **b,** After wholemount imaging clone YP.1 was embedded and sections were cut > 25 μm apart to a depth of approximately 300 μm. All EYFP$^+$ cells expressed K8 and were negative for SMA. DAPI was used to label all nuclei. Images are representative of 10 sections comprising over 281 EYFP$^+$ cells from a single clone (summarised in Fig. 3).

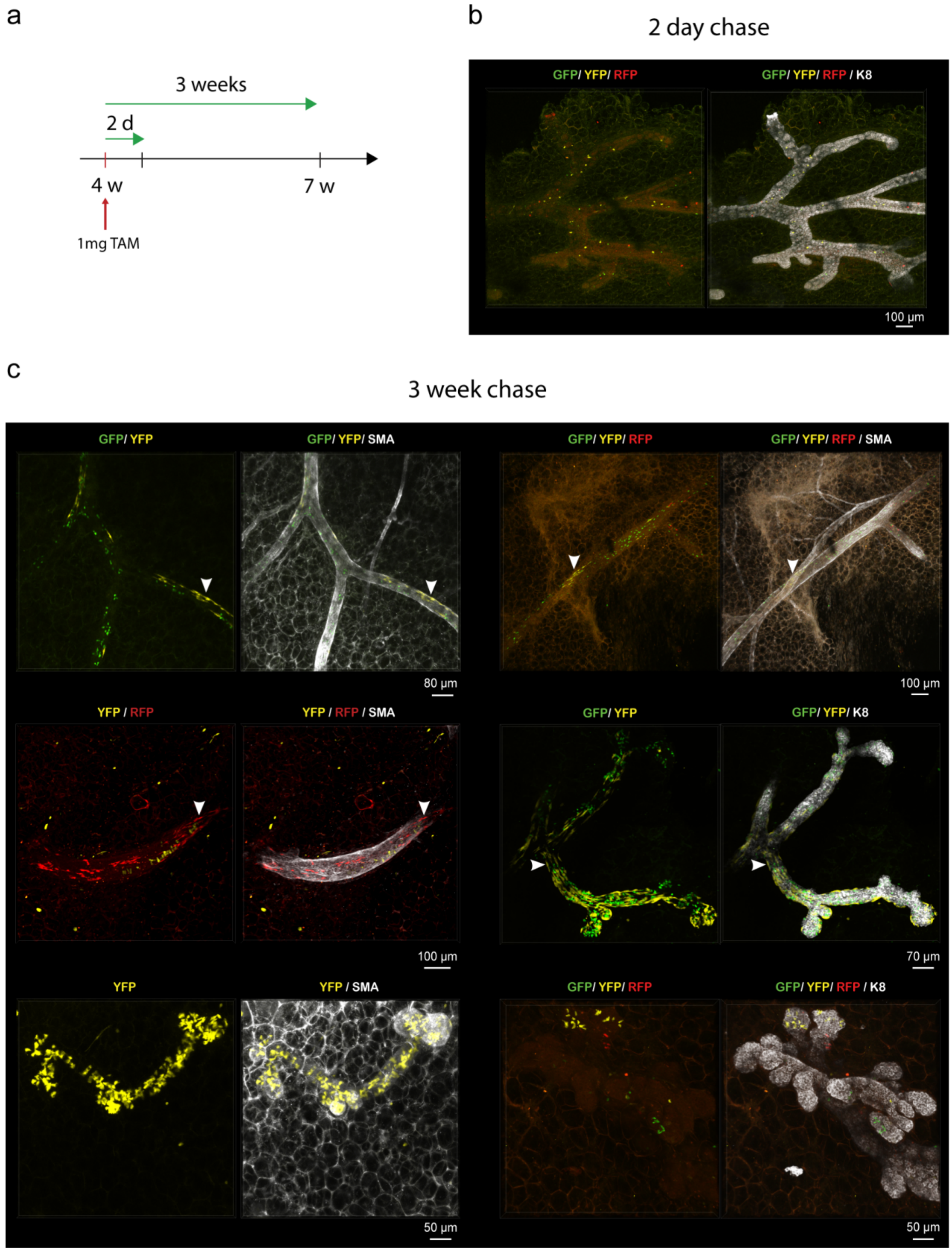

Supplementary Figure 11: Examples of the labelling patterns observed in a neutral multicolour lineage-tracing model related to Figure 5. a, Schematic representation of the tamoxifen induction protocol in *R26-Confetti;R26-CreERT2* mice. **b**, Initial labelling level observed after a 2-day chase. **c**, Labelling patterns observed in *R26-Confetti;R26-CreERT2* mice after a 3 week chase during puberty, basal regions are shown (arrowhead).

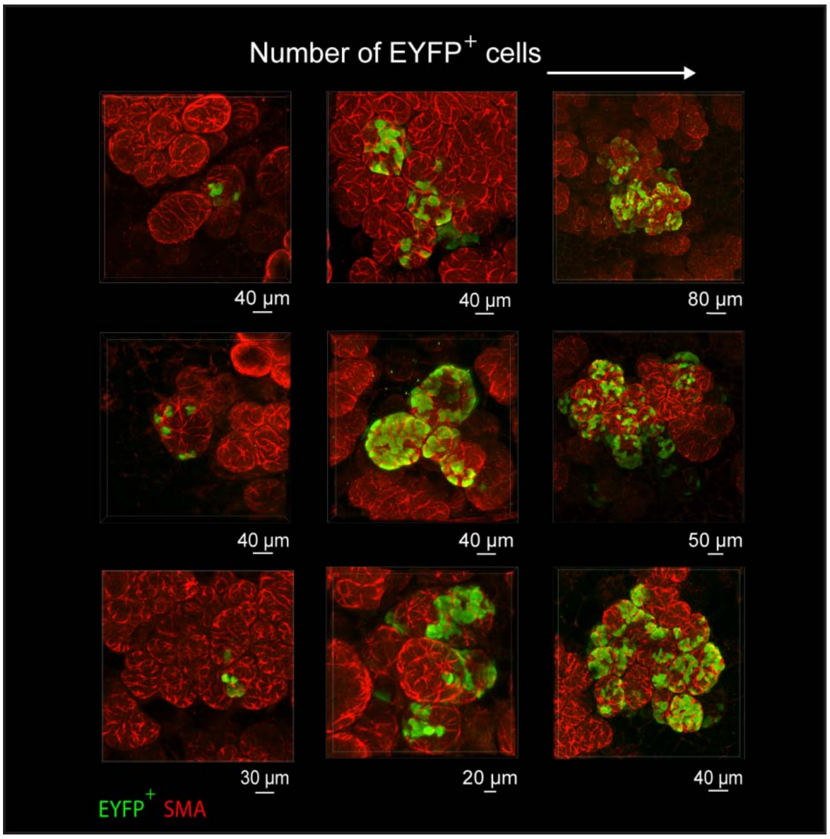

Supplementary Figure 12: Examples of EYFP^{+} regions of various sizes observed in lactating *R26$^{[CA]30EYFP}$* mice.

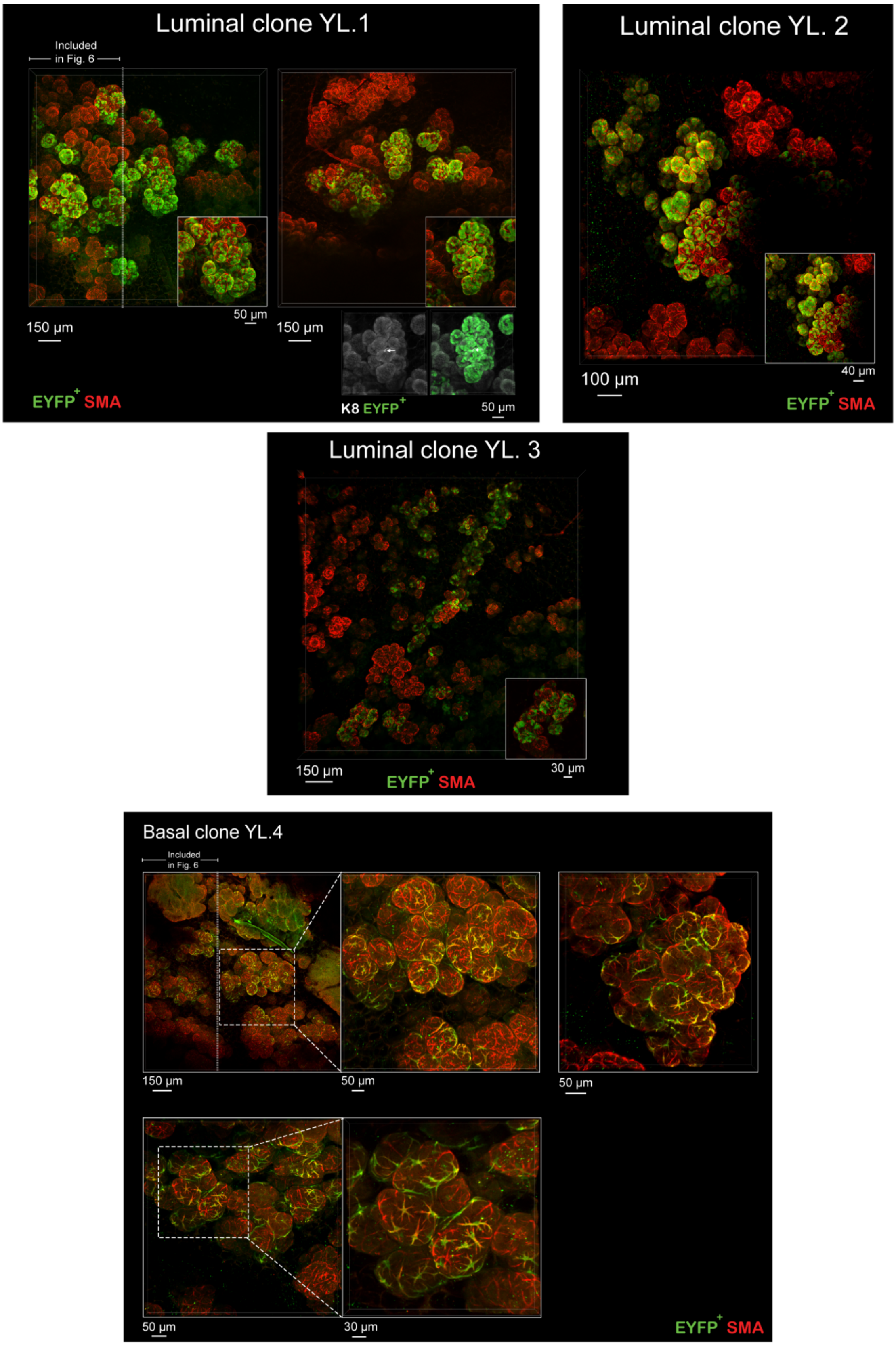

Supplementary Figure 13: Clone size and labelling patterns arising from a single EYFP^{+} cell in lactating mammary glands related to Figure 6. Wholemount confocal images of independent luminal (YL.1, YL.2 and YL.3) and basal (YL.4) EYFP^{+} clones, revealing the extensive contribution of one labelled cell to the mammary lobuloalveolar network. Inset below the luminal clone YL.1 shows the contribution of EYFP^{+} cells to both K8^{lo} cells and a rare K8^{hi} cell (arrow). Further regions from these clones are shown in Fig. 6.

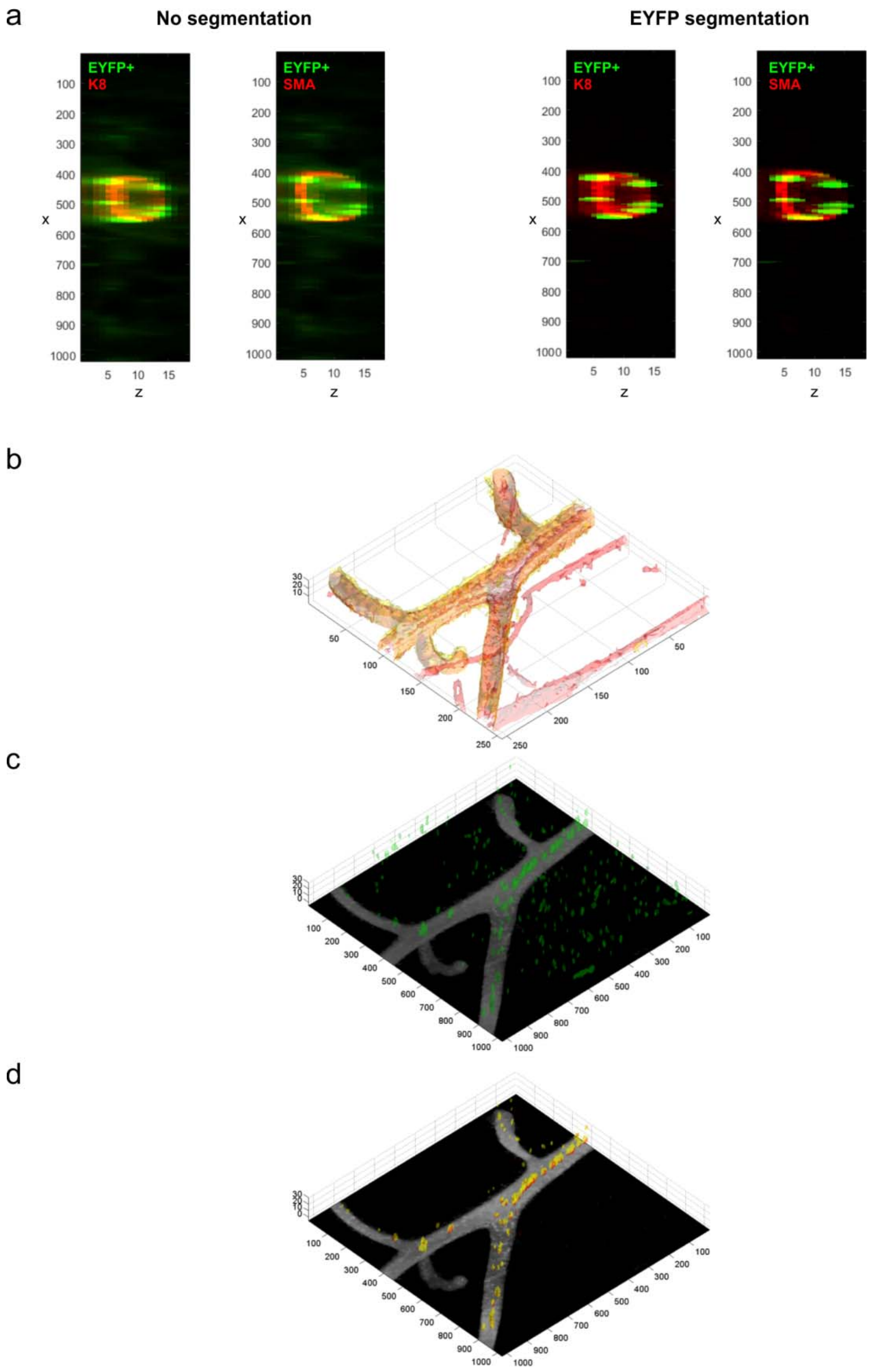

Supplementary Figure 14: Optical resolution through z and 3D image analysis of clones. Methods for segmentation and classification (see Methods).
